# Annotations

**Published:** 1896-12-05

**Authors:** 


### ANNOTATIONS.

### Pneumatic Cushions for Sleeping Cars.

AN innovation in regard to the cushions for railway carriages and the bedding for sleeping cars is reported from America, which, if it is found to be feasible in practice on the large scale, is likely to be of very great service to railway travellers. One great cause of nervous disturbance among travellers by rail is the rapid flashing of external objects past the eyes, connected with which also is the fatigue of the eye muscles, resulting from the attempt to follow the moving objects, and from the rapid changes of accommodation involved in doing so. As a result of all this, headache of a truly neuralgic or migrainous type often results. The cure, of course, is to pull down the blinds. Vibration is another matter which is far from being so easily dealt with. A car has, however, been recently fitted up with pneumatic cushions instead of those of the ordinary type, and the result is said to be extremely good. Knowing travellers have long been in the habit of providing themselves with air cushions, so as to save their spines from vibration and get some rest for their heads, and it is said that when seated on the pneumatic cushions now introduced, a marked difference is experienced in comparison with the ordinary carriage seats. It is not, however, in the saving of vibration alone that these india-rubber air beds and cushions are expected to be useful. It is hoped that they will largely solve a difficulty which has long stood in the way of an extended application of the sleeping car system, viz., that of converting the car to other purposes in consequence of the space occupied by the bedding. On the pneumatic system this difficulty vanishes, for when the air is let out the bedding can be packed away into the smallest space; in fact, it all shuts up against the side, and the carriage can be used for ordinary traffic. The wear and tear of the human frame produced by railway travelling is very considerable, and any means by which one of its main trials—that is, vibration—can be diminished, ought to increase the number of those who fly to the country so soon as their work is done.

### The Torbay Hospital.

THE authorities of the Torbay Hospital, at Torquay, seem to have been considerably upset by a complaint made by the Newton Abbot Board of Guardians to the effect that certain applicants for admission to the hospital had been refused on the ground that they were paupers. Not only does the secretary of the hospital write to say that it is not the fact that the patient in question was refused admission because he was a pauper, but the chairman of the Weekly Board writes to assure the Guardians that, during his many years' experience of the work of the hospital, no one has ever been refused admittance simply and purely on the ground of his being in receipt of parish relief. As the local papers say, a "complete answer" has been given to the "charges" made against the hospital. Still, the charges do not appear to us to have been particularly serious, and we are not certain that the defence is not as suggestive of weakness of administration as were the charges. The broad statement that the hospital is for the poor seems to be taken by some as a complete vindication of the practice of admitting paupers to its benefits. The fact is, however, that paupers are not poor in a technical sense, for they have the rates at the back of them. The Guardians subscribe £21 per annum to the hospital, and so far as that subscription covers the expense arising from the reception of paupers it may be right to receive them. But in no other way can it be accepted as an axiom of hospital management that paupers are eligible as patients. The Guardians are bound by law to provide for the paupers who come under their care, and unless the Guardians pay for all the expenses that a voluntary hospital is put to in the treatment of any paupers that may be admitted, the hospital funds are being diverted from their proper purpose and used for the lessening of the poor-rate—which is certainly not the object for which they were subscribed; even a liberal subscription of £21 per annum does not go far. In Burdett's "Hospitals and Charities" the average annual cost per bed occupied is put at about £75, so that the subscription above-mentioned would only cover the use of a bed for about fourteen weeks in the year.

### The Physiological Action of German Bands.

ENVIRONMENT, as is well known, is one of the most potent of all the modifying circumstances known to animal and vegetable life. Everything to which man is in any way, consciously or unconsciously, but actually related, has a more or less powerful physiological action upon his organism. "South Kensington," writing to the *Times*, gives an account, which can however by no means be complete, of some of the physiological effects produced upon certain human organisms in his neighbourhood by the playing of German bands. The effects, as may be readily imagined, were of at least two kinds, physical and mental. It is more than probable that in some cases, at any rate, effects of a moral kind were also produced. One of the earliest physical consequences was that of rapid motor action on the part of the householder. He "sallied out," we are told, at a dangerous pace, to "call the police." A second effect was the "precluding of all attempts to work, or read, or write, or even rest." "Rest," in fact, seems to have been the remotest of all possibilities under the circumstances. In the case of children, very unfortunate consequences are reported; they "could not sleep"; and to outdoor "braying" was added indoor "wailing," with results to the grown-up victims which "South Kensington" wisely refrains from attempting to describe. Students reading for competitive examinations are said to have been driven, first to despair, and then from London altogether, to seek that physiological repose which a too-Germanised metropolis so ruthlessly denied them. The more purely mental and moral effects we have not space to enter upon. The least imaginative may picture them for himself. It is the duty of THE HOSPITAL to heal, and to teach the healing art. We would fain help to heal the hurts of "South Kensington" and his neighbours. According to modern scientific medicine the most sovereign of all known panaceas is to "remove the cause." That is the panacea the use of which we commend to South Kensington; and we advise that it be applied with all possible promptitude and energy if further and still worse evils are to be averted.

